# 30-year experience of Fontan surgery: single-centre's data

**DOI:** 10.1186/1749-8090-10-S1-A121

**Published:** 2015-12-16

**Authors:** Laurynas Bezuska, Virgilijus Lebetkevicius, Kestutis Lankutis, Rita Sudikiene, Daina Liekiene, Vytautas J Sirvydis, Virgilijus Tarutis

**Affiliations:** 1 Department of Cardiovascular Medicine, Vilnius University, Vilnius, 08661, Lithuania; 2 Vilnius University Hospital Santariskiu Klinikos, Centre of Cardiac Surgery, Vilnius, 08661, Lithuania

## Background/Introduction

The Fontan procedure has been modified several times since it was introduced into practice in 1968. As many patients now survive to adulthood, attention is directed towards their clinical status and late morbidities.

## Aims/Objectives

We report our surgical experience of 30 years in Fontan procedures and the long-term follow-up result.

## Method

From January 1985 to January 2015, 80 patients underwent Fontan surgery. Twenty one patient received an atrio-pulmonary Fontan (Group I), 4 patients underwent total cavopulmonary connection (TCPC) with an intra-atrial lateral tunnel (Group II), 6 patients received extra-cardiac TCPC with an aortic homograft (group III) and 49 patients received extra-cardiac TCPC with an expanded polytetrafluoroethylene conduit. They were followed for early and late mortality, long-term survival, postoperative morbidity and reintervention or reoperation.

## Results

The mean follow-up time was 7.4 ± 6.6 years. The Kaplan-Meier estimated 15-year survival rate was 42% in Group I, 50% in Group II, 83% in Group III and 94% in Group IV. The median preoperative pulmonary artery pressure and pulmonary artery resistance were 13 mm Hg (IQR, 11-15) and 1 Wood unit (IQR, 1-1.4) respectively. Fenestration was created in 40 (50%) patients. The median length of stay in intensive care unit, intubation and chest drain stay time were 90 hours (interquartile range [IQR], 46-119), 8 hours (IQR, 6-16) and 18 days (IQR, 12-28) respectively. Early complications were bleeding in 6, taken down of Fontan circulation in 3 and acute heart failure managed by left heart bypass in 1 patient. Late-occurring morbidities included arrhythmias in 6, protein-losing enteropathy in 2, thromboembolism in 2 and tracheal stenosis in 1 patient. Fourteen patients (18%) had redo Fontan procedures.

## Discussion/Conclusion

Our series showed improving results after Fontan completion with excellent mid-term outcome after extra-cardiac TCPC with expanded polytetrafluoroethylene conduit. The long-term result should be followed.

